# Protective Role of Matrix Metalloproteinase-9 in Ozone-Induced Airway Inflammation

**DOI:** 10.1289/ehp.10289

**Published:** 2007-08-24

**Authors:** Hyoung-Kyu Yoon, Hye-Youn Cho, Steven R. Kleeberger

**Affiliations:** 1 Laboratory of Respiratory Biology, National Institute of Environmental Health Sciences, National Institutes of Health, Department of Health and Human Services, Research Triangle Park, North Carolina, USA; 2 Division of Pulmonary and Critical Care, Department of Internal Medicine, College of Medicine, Catholic University of Korea, Seoul, Korea

**Keywords:** chemokine, knockout mice, lung, MMP-9, O_3_, oxidant

## Abstract

**Background:**

Exposure to ozone causes airway inflammation, hyperreactivity, lung hyper-permeability, and epithelial cell injury. An early inflammatory response induced by inhaled O_3_ is characterized primarily by release of inflammatory mediators such as cytokines, chemokines, and airway neutrophil accumulation. Matrix metalloproteinases (MMPs) have been implicated in the pathogenesis of oxidative lung disorders including acute lung injury, asthma, and chronic obstructive pulmonary disease.

**Objective:**

We hypothesized that MMPs have an important role in the pathogenesis of O_3_-induced airway inflammation.

**Methods:**

We compared the lung injury responses in either *Mmp7*- (*Mmp7*^−/−^) or *Mmp9*-deficient (*Mmp9*^−/−^*)* mice and their wild-type controls (*Mmp7^+/+^, Mmp9^+/+^)* after exposure to 0.3 ppm O_3_ or filtered air.

**Results:**

Relative to air-exposed controls, MMP-9 activity in bronchoalveolar lavage fluid (BALF) was significantly increased by O_3_ exposure in *Mmp9^+/+^* mice. O_3_-induced increases in the concentration of total protein (a marker of lung permeability) and the numbers of neutrophils and epithelial cells in BALF were significantly greater in *Mmp9*^−/−^ mice compared with *Mmp9^+/+^* mice. Keratinocyte-derived chemokine (KC) and macrophage inflammatory protein (MIP)-2 levels in BALF were also significantly higher in *Mmp9*^−/−^ mice than in *Mmp9^+/+^* mice after O_3_ exposure, although no differences in mRNA expression for these chemokines were found between genotypes. Mean BALF protein concentration and numbers of inflammatory cells were not significantly different between *Mmp7^+/+^* and *Mmp7*^−/−^ mice after O_3_ exposure.

**Conclusions:**

Results demonstrated a protective role of MMP-9 but not of MMP-7, in O_3_-induced lung neutrophilic inflammation and hyperpermeability. The mechanism through which *Mmp9* limits O_3_-induced airway injury is not known but may be via posttranscriptional effects on proinflammatory CXC chemokines including KC and MIP-2.

Ozone is a common urban air pollutant that remains a major health concern. Epidemiologic studies have demonstrated a strong association between high ambient O_3_ concentration with cardiovascular and respiratory morbidity and mortality (Bell et al. 2005). O_3_ exposure elicits airway inflammation characterized by neutrophil accumulation (Schelegle et al. 1991; Seltzer et al. 1986) and liberates multiple inflammatory mediators, cytokines, and chemokines as an early inflammatory event (Aris et al. 1993; Koren et al. 1989). O_3_-induced activation of airway neutrophilic infiltration is likely to produce additional damage through the release of reactive oxygen species and endogenous proteolytic enzymes.

Several different kinds of endogenous proteases participate in the pathogenesis of airway inflammation. Among these, matrix metalloproteinases (MMPs) belong to a family of zinc-dependent extracellular enzymes that share common structural features and have an important role in many physiological and pathological conditions (Parks and Shapiro 2001). MMPs are secreted by inflammatory and noninflammatory cells and have a major role in extracellular matrix (ECM) degradation, proteolytic modulation of biologically active proteins, and cell migration (Goetzl et al. 1996; Kelly et al. 2000; Kheradmand et al. 2002; Mautino et al. 1997; Parks and Shapiro 2001). Elevated levels of MMPs are prominent in inflammatory disorders of the airway and parenchymal lung disease and have been also implicated in several pulmonary diseases characterized by lung tissue damage, alveolar structure alterations, or abnormal repair (Elkington and Friedland 2006; Parks and Shapiro 2001). MMPs can be divided on the basis of their substrate specificity into collagenases (MMP-1, 8, 13), gelatinases (MMP-2, 9), stromelysins (MMP-3, 10, 11), matrilysin (MMP-7), macrophage metalloelastase (MMP-12), and membrane-type (MT)-MMPs (MMP-14, 15, 16, 17) (Visse and Nagase 2003).

Gelatinases degrade many ECM proteins; most important is type IV collagen, a major constituent of lung basement membrane (Nagase and Woessner 1999). Therefore, gelatinases have been thought to have an important role in the pathogenesis of various lung diseases (Nagase and Woessner 1999). Gelatinases also can cleave a variety of non-ECM proteins, including cytokines and chemokines (McQuibban et al. 2000; Yu and Stamenkovic 2000; Zhang et al. 2003). MMP-2 (gelatinase A) is produced by a wide range of cell types in the lung, and it is increased in lung disorders with oxidative stress and inflammation etiologies (e.g., Pardo et al. 1998; Perez-Ramos et al. 1999; Tan et al. 2006). MMP-9 (gelatinase B) has been implicated in the pathogenesis of asthma (Cataldo et al. 2000), idiopathic pulmonary fibrosis (Fukuda et al. 1998), chronic obstructive pulmonary disease (COPD; Russell et al. 2002), and acute lung injury (Lanchou et al. 2003). In the normal lung, constitutive expression of MMP-9 is restricted to neutrophils and eosinophils. However, MMP-9 induced by inflammatory stimuli and proinflammatory cytokines is generated in multiple cell types including alveolar macrophages and other resident pulmonary cells (Atkinson and Senior 2003; Chakrabarti and Patel 2005). MMP-9 is known to be induced by O_3_ in murine skin and lung (Kenyon et al. 2002; Valacchi et al. 2003).

MMP-7 has homeostatic function and a role in innate immunity in epithelial cells (Parks and Shapiro 2001). In adult human lung, MMP-7 is expressed by epithelial cells lining peribronchial and conducting airway (Dunsmore et al. 1998). Increased expression of MMP-7 has been observed in airway and alveolar cells at sites of lung cancer (Wilson and Matrisian 1996), idiopathic pulmonary fibrosis (Zuo et al. 2002), and cystic fibrosis (Dunsmore et al. 1998). Although it is clear that MMP-7 is associated with chronic lung disease, the role of MMP-7 in O_3_-induced airway inflammation is still unknown.

In the present studies, we hypothesized that murine MMP-7 and MMP-9 have important functional roles in facilitating the airway inflammatory responses induced by inhaled O_3_. To address this hypothesis, we used mice genetically deficient in *Mmp7* (Mmp7, matrix metalloproteinase 7; Entrez Gene ID 17393, http://www.ncbi.nlm.nih.gov/sites/entrez?db=gene) and *Mmp9* (Mmp9; matrix metalloproteinase 9; Entrez Gene ID 17395, http://www.ncbi.nlm.nih.gov/sites/entrez?db=gene) and compared airway injury responses with O_3_ relative to corresponding wild-type control mice.

## Materials and Methods

### Animals and inhalation exposure

Male (6–8 weeks old, 20–25 g) C57BL/6J (*Mmp7^+/+^*) and B6.129-*Mmp7^tm1Lmm^*/J (*Mmp7*^−^*^/^*^−^), FVB/NJ (*Mmp9^+/+^*), and FVB.Cg-*Mmp9^tm1Tvu^*/J (*Mmp9*^−^*^/^*^−^) mice were purchased from The Jackson Laboratory (Bar Harbor, ME). Inhalation exposures were performed in a barrier facility at Alion Science and Technology, Inc. (Research Triangle Park, NC). After acclimation, mice were placed in individual stainless-steel wire cages within a Hazelton 1000 chamber (Lab Products, Maywood, NJ) equipped with a charcoal and high-efficiency particulate air-filtered air supply. Mice had free access to water and pelleted open-formula rodent diet NIH-31 (Zeigler Bros, Inc., Gardners, PA). Mice were exposed continuously to 0.3 ppm O_3_ for 6, 24, 48, or 72 hr. O_3_ was generated from ultra-high purity air (< 1 ppm total hydrocarbons; National Welders, Inc., Raleigh, NC) using a silent arc discharge O_3_ generator (Model L-11; Pacific Ozone Technology, Benecia, CA). Constant chamber air temperature (72 ± 3°F) and relative humidity (50 ± 15%) were maintained. O_3_ concentration was continually monitored (Dasibi model 1008-PC, Dasabi Environmental Corp., Glendale, CA). Parallel exposure to filtered air was conducted in a separate chamber for 48 hr. All animal use was approved by the National Institute of Environmental Health Sciences Animal Care and Use Committee, and all animals were treated humanely and with regard for alleviation of suffering.

### Lung bronchoalveolar lavage fluid (BALF) and cell preparation

Immediately after exposure to air or O_3_, mice were euthanized with sodium pentobarbital (104 mg/kg). After 24-or 48-hr exposure, BAL was performed *in situ* 4 times with Hanks balanced salt solution (35 mL/kg; pH 7.2–7.4). These time points were chosen for BAL procedures on the basis of previous investigations with multiple inbred strains of mice that indicated inflammation and injury peaked between 24 and 48 hr during continuous exposure to 0.3 ppm O_3_ (e.g., Cho et al. 2007; Dahl et al. 2007; Kleeberger et al. 1993). Recovered BALF was immediately cooled in ice and centrifuged (1,000 × *g*, 10 min). The cell pellets were resuspended in 1 mL of Hanks balanced salt solution, and the cells were counted with a hemacytometer. Aliquots (150 μL) were cyto-centrifuged, and the cells were stained with Wright-Giemsa stain solutions (Fisher Scientific, Pittsburgh, PA) for differential cell analysis (markers of cellular inflammation and epithelial injury). The supernatants from the first BALF return were assayed for total protein (a marker of lung permeability) with the Bradford assay (Bradford 1976).

### Lung tissue preparation for histopathology and immunohistochemistry

Left lung lobes from mice exposed to either 0.3 ppm O_3_ or filtered air for 48 hr were inflated gently with zinc formalin, fixed at a constant pressure (25 cm) of the same fixative for 30 min, tied off at the trachea, and immersed in the fixative for 1 day. Proximal and distal intrapulmonary axial airway was excised, embedded in paraffin, and sectioned (5 μm). Tissue sections were histochemically stained with hematoxylin and eosin (H&E) for morphological comparison of pulmonary injury between genotypes. The terminal bronchioles and alveoli were the primary focus of study because 48-hr exposure to 0.3 ppm O_3_ causes histologically evident inflammation and epithelial lesions in these regions of the mouse lung. Separate tissue sections from *Mmp9^+/+^* or *Mmp9*^−^*^/^*^−^ mice were immunologically stained using a goat anti-mouse MMP-9 antibody (R&D Systems, Inc., Minneapolis, MN) or a rat anti-mouse Ki-67 antibody (Dako North American, Inc., Carpinteria, CA) to localize MMP-9 and Ki-67, respectively, using the standard peroxidase–diaminobenzidine (DAB) method.

### Western blot analysis

Equal amounts of total lung protein (45 μg) isolated from mouse lung homogenates (air or 24, 48, and 72 hr of O_3_) in radioimmunoprecipitation (RIPA) buffer (1% Nonidet P-40, 0.5% sodium deoxycholate, 0.1% sodium dodecyl sulfate (SDS), 10 g phenylmethylsulfonyl fluoride/mL, 1 mM sodium orthovanadate, 1× protease inhibitor cocktail in 1× phosphate-buffered saline) were separated on 10–20% SDS gel electrophoresis gels. The 24-, 48-, and 72-hr time points were chosen for investigation to characterize the kinetics of lung MMP activity changes induced by O_3_ exposure. Proteins were then transferred to a polyvinylidene fluoride (PVDF) membrane. The membrane was blocked for 2 hr at room temperature with 5% nonfat milk in Tris-buffered saline–Tween buffer (20 mmol/L Tris, 500 mmol/L NaCl, 0.01% Tween 20). The blot was then incubated at 4°C overnight with anti-MMP-9 (R&D Systems), anti-MMP-2 (R&D Systems), or an anti-actin antibody (SantaCruz Biotechnology Inc., Santa Cruz, CA) as an internal control, followed by incubation for 1–2 hr with the proper secondary horseradish peroxidase-conjugated antibodies. The immunoblot was visualized through enhanced chemiluminescence.

### Total RNA isolation and real-time quantitative reverse transcriptase–polymerase chain reaction (RT-PCR)

Total RNA from frozen lungs (air or 6, 24, and 48 hr of O_3_) was isolated using the RNeasy Midiprep kit (QIA-GEN, Valencia, CA). The 6-hr time point was included in this protocol to optimize the likelihood of capturing early inflammatory cytokine/chemokine gene expression on the basis of previous experience with this model (Cho et al. 2007). One microgram total RNA was reverse transcribed into cDNA in a volume of 50 μL containing 1× PCR buffer [50 mM KCl and 10 mM Tris (pH 8.3)], 5 mM MgCl_2_, 1 mM each dNTPs, 125 ng oligo (dT)_15_, and 50 U of Moloney murine leukemia virus reverse transcriptase (Invitrogen Life Technologies, Carlsbad, CA), at 45°C for 15 min and 95°C for 5 min using the Gene Amp PCR System 9700 (Applied Biosystems, Foster City, CA).

An aliquot (2–5 μL) of the reverse transcriptase product (equivalent to 40–100 ng RNA) was amplified using PowerSYBR gene expression assays on the 7700 Prism sequence detection system (Applied Biosystems). The PCR conditions and data analysis were performed according to the manufacturer’s protocol in User Bulletin no. 2, “Applied Biosystems Prism 7700 Sequence Detection System” (Applied Biosystems 2007). Quantification of gene expression was determined by the number of cycles to threshold (C_T_) of fluorescence detection. Relative gene expression was determined using the comparative C_T_ method as described previously (Chakrabarti and Patel 2005). Briefly, the ΔC_T_ value was obtained by subtracting the 18s rRNA C_T_ value from the C_T_ value of the gene tested in the same sample. Message levels of each gene were expressed as fold changes relative to those in *Mmp9^+/+^* air controls.

### Gelatin zymography analysis

Equal volumes of BALF (10 μL) samples were loaded on 10% Tris–glycine gel with 0.1% gelatin (Invitrogen Life Technologies) for electrophoresis. To eliminate the SDS content, the gels were washed with 2.7% (wt/vol) Triton X-100 at room temperature for 30 min and incubated for 16 hr in a buffer containing 50 mM Tris base, 40 mM HCl, 200 mM NaCl, 5 mM CaCl_2_, and 0.02% (wt/vol) Brij 35 for gelatinolytic enzymes to act. The digested gels were stained in Colloidal Blue Staining kit (Bio-Rad Laboratories, Hercules, CA) for 4 hr to visualize the gelatin activity. Gelatinolytic bands were analyzed by a Gel Doc 2000 System (Bio-Rad).

### Cytokine enzyme-linked immunosorbent assay (ELISA)

Immunoreactive macrophage inflammatory protein-2 [MIP-2, human interleukin-8 (IL-8) analogue, similar to chemokine (C-X-C motif) ligand (CXCL) 1 and 2] and keratinocyte-derived chemokine [KC, human growth-regulated oncogene (GRO)-α analogue, similar to CXCL1, 2, and 3] were quantified in aliquots of BALF (50 μL) using commercially available ELISA kits (R&D Systems), according to the manufacturer’s instructions. Each cytokine quantity was calculated from absorbance at 450 nm using a standard curve.

### Statistics

All data are expressed as group means ± SE. Two-way analysis of variance (ANOVA) was used to evaluate the effects of subacute O_3_ exposure on lung injury parameters between *Mmp7^+/+^* and *Mmp7*^−^*^/^*^−^ or *Mmp9^+/+^* and *Mmp9*^−^*^/^*^−^ mice. The factors in the analysis were exposure (air vs. O_3_) and genotype (*Mmp7^+/+^* vs. *Mmp7*^−/−^*, Mmp9^+/+^* vs. *Mmp9*^−/−^). The dependent variables were protein level, cell number, MMP-2 activity, and cytokine concentrations in BALF, and lung protein and mRNA levels. One-way ANOVA was used to evaluate the effect of O_3_ on MMP-9 production and expression in *Mmp9^+/+^* mice. The Student–Newman–Keuls test was used for *a posteriori* comparisons of means. All analyses were performed using a commercial statistical analysis package (SigmaStat; SPSS Inc., Chicago, IL). Statistical significance was accepted at *p* < 0.05.

## Results

### Roles of MMP-7 and MMP-9 in O_3_-induced airway inflammation

**Figure 1:**
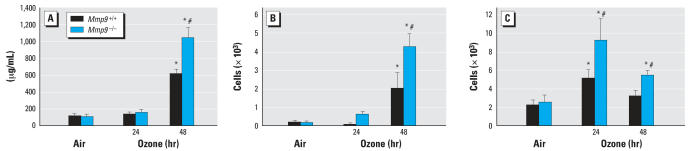
Concentration of total protein (*A*) and numbers of neutrophils (*B*) and epithelial cells (*C*) in BALF recovered from *Mmp9^+/+^* and *Mmp9*^−^*^/^*^−^ mice after exposure to 0.3 ppm O_3_ or filtered air. Data are presented as means ± SE (*n* = 8–10/group). *Significantly different from genotype-matched air control mice (*p* < 0.05). ^#^Significantly different from exposure-matched *Mmp9^+/+^* mice (*p* < 0.05).

The roles of MMP-7 and MMP-9 in O_3_-induced airway inflammation were assessed by exposing *Mmp7^+/+^, Mmp7*^−^*^/^*^−^*, Mmp9^+/+^,* and *Mmp9*^−^*^/^*^−^ mice continuously to air or O_3_ for 24 and 48 hr. No significant differences in mean BAL protein concentration or cell numbers were found between respective +/+ and −*/*− genotypes after exposure to air (Figure 1, Table 1). After 48 hr of O_3_ exposure, BAL protein concentration was significantly (*p* < 0.05) increased in all four genotypes (Figure 1, Table 1). The BALF protein concentration was significantly greater (68%) in *Mmp9*^−^*^/^*^−^ mice compared with *Mmp9^+/+^* mice (Figure 1A); however, no significant differences in protein concentration were found between *Mmp7^+/+^* and *Mmp7*^−^*^/^*^−^ mice after exposure to O_3_ (Table 1). O_3_ caused significant increases in the mean numbers of BAL neutrophils in *Mmp9^+/+^* and *Mmp9*^−^*^/^*^−^ mice after 48 hr of O_3_ exposure (*p* < 0.05; Figure 1B). However, compared with *Mmp9*^+/+^ mice, O_3_-enhanced BAL neutrophil numbers were significantly higher in *Mmp9*^−^*^/^*^−^mice (*p* < 0.05). Mean numbers of epithelial cells were also significantly greater in *Mmp*^−^*^/^*^−^ mice relative to those in *Mmp^+/+^* mice after 24- and 48-hr O_3_ exposure (*p* < 0.05; Figure 1C). No significant differences in the number of BALF alveolar macrophages were found between *Mmp9^+/+^* and *Mmp9*^−/−^ mice (data not shown). No significant differences in mean numbers of BALF inflammatory cells were found between *Mmp7*^−/−^ and *Mmp7^+/+^* mice after exposure to O_3_ (Table 1).

### Effect of O_3_ on MMP-2 and MMP-9 expression in the lung

**Figure 2:**
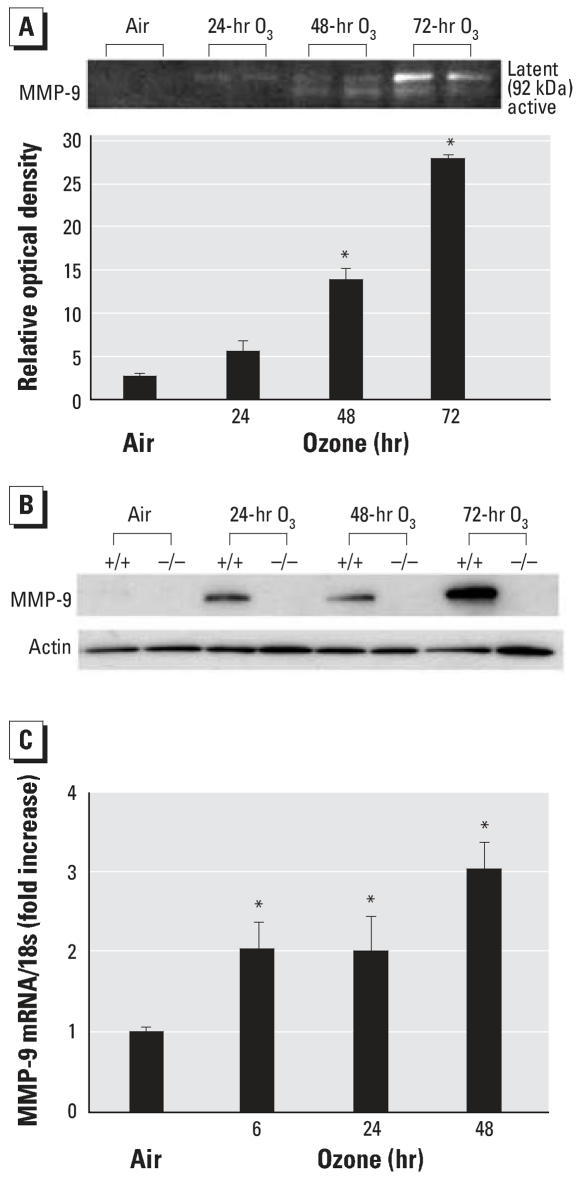
MMP-9 mRNA, protein, and activity after exposure to 0.3 ppm O_3_ or filtered air. (*A*) Representative images of MMP-9 activity in BALF from *Mmp9^+/+^* mice determined by gelatin zymogram after exposure to 0.3 ppm O_3_ (24, 48, 72 hr) or filtered air (*n* = 3/group). (*B*) Representative Western blots of MMP-9 in pooled lung homogenates from *Mmp9^+/+^* (+/+) and *Mmp9*^−/−^ (−/−) mice after exposure to 0.3 ppm O_3_ (24, 48, 72 hr) or filtered air (*n* = 3/group). (*C*) MMP-9 mRNA expression in lung homogenates from *Mmp9^+/+^* mice after exposure to 0.3 ppm O_3_ (6, 24, 48 hr) or filtered air. Quantitative real-time PCR-determined fold increase of normalized expression to 18s rRNA compared with air-exposed mice (*n* = 3/group). Data are presented as means ± SE. *Significantly different from air control mice (*p* < 0.05).

**Figure 3:**
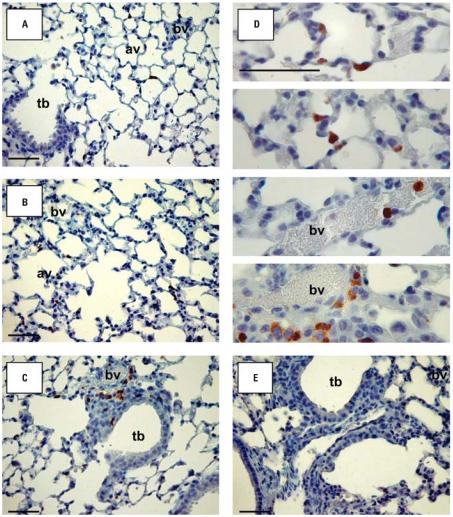
Immunohistochemical localization of MMP-9 in the lungs of *Mmp9^+/+^* mice after filtered air (*A*) and 48-hr O_3_ (*B,C,D*) exposure. Representative light micrographs at higher magnifications (*D*) show MMP-9–positive cells (in brown) including alveolar epithelial cells, infiltrating inflammatory cells, myofibroblasts, and cells in the injured regions undergoing proliferation and differentiation. Lung tissue sections from *Mmp9*^−/−^ mice exposed to O_3_ (*E*) were similarly processed and visualized as negative controls. Abbreviations: av, alveoli; bv, blood vessels; tb, terminal bronchioles. Bars = 50 μm.

We performed gelatin zymogram and Western blot analyses on BALF and lung protein extracts from *Mmp9^+/+^* and *Mmp9*^−^*^/^*^−^ mice to determine whether production of MMP-2 and MMP-9 was augmented by O_3_ exposure. Both gelatinases have been implicated in the pathogenesis of inflammation-related lung diseases (e.g., Pardo et al. 1998; Tan et al. 2006); we also reasoned that MMP-2 expression might be enhanced to compensate for MMP-9 deletion. Minimal gelatinase activity was found in BALF from either genotype after air exposure. Exposure to O_3_ (48 and 72 hr) caused significant (*p* < 0.05) increase in MMP-9 activity at 92 kDa (latent form) and 88 kDa (active form) in BALF from *Mmp9^+/+^* mice (Figure 2A). Western blot analyses confirmed O_3_-induced increase in MMP-9 protein levels in lung homogenates from *Mmp9^+/+^* mice (Figure 2B). Furthermore, lung MMP-9 mRNA expression in *Mmp9^+/+^* mice was significantly increased compared with air controls after 6 hr of O_3_ and remained increased by 48-hr exposure (*p* < 0.05; Figure 2C). MMP-9 proteins were localized primarily in alveolar epithelial cells, infiltrating inflammatory cells, endothelial cells, and cells in the injured parenchyma of *Mmp9^+/+^* mice after O_3_ exposure (Figure 3).

**Figure 4:**
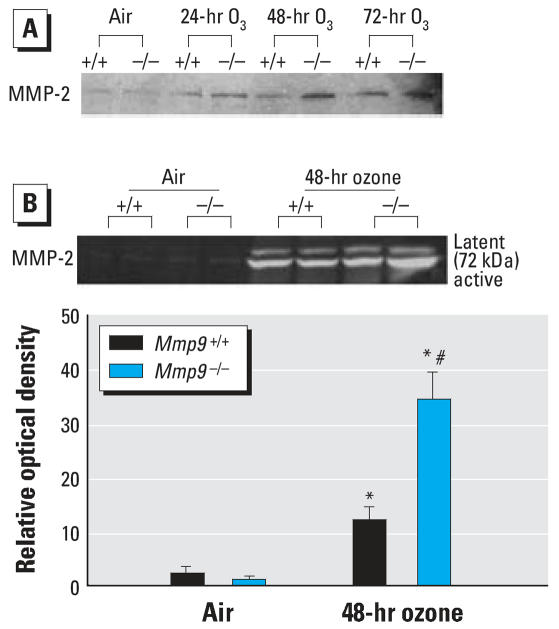
Measurement of MMP-2 in BALF and lung protein extracts from *Mmp9^+/+^* and *Mmp9*^−/−^ mice after exposure to filtered air or 0.3 ppm O_3_. (*A*) Representative Western blot image of MMP-2 protein expression in pooled lung homogenates from *Mmp9^+/+^* (+/+) and *Mmp9*^−/−^ (−/−) mice after exposure to 0.3 ppm O_3_ (24, 48, 72 hr) or filtered air (*n* = 3/group). (*B*) Representative gelatin zymogram image for MMP-2 activity in BALF from *Mmp9^+/+^* (+/+) and *Mmp9*^−/−^ (−/−) mice after exposure to 0.3 ppm O_3_ (48 hr) or filtered air. Relative optical density of MMP-2 activity quantified from zymogram blots. Data are presented as means ± SE (*n* = 3/group). *Significantly different from genotype-matched air control mice (*p* < 0.05). ^#^Significantly different from O_3_ (48 hr)-exposed *Mmp9^+/+^* mice (*p* < 0.05).

Basal levels of MMP-2 protein and activity were detectable in lung protein extract and BALF, respectively, from *Mmp9^+/+^* and *Mmp9*^−^*^/^*^−^ mice, although no differences were detected between genotypes. O_3_ increased MMP-2 activity and production in both genotypes compared with corresponding baseline (Figure 4A,B), but the increases were significantly (*p* < 0.05) greater in *Mmp9*^−/−^ compared with *Mmp9^+/+^* mice.

### *Effects of* Mmp9 *deficiency on O_3_-induced pulmonary pathology.*

**Figure 5:**
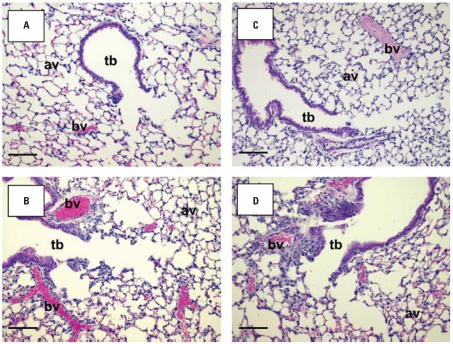
Lung histology from *Mmp9^+/+^* (*A,B*) and *Mmp9*^−/−^ (*C,D*) mice exposed to air (*A,C*) and 48 hr O_3_ (*B,D*). Paraffin-embedded left lung tissue sections were stained with H&E for histopathologic analysis. Representative light micrographs show more severe epithelial thickening, inflammation, and alveolar vacuolization in distal airways of *Mmp9*^−/−^ mice (*D*) relative to those of *Mmp9^+/+^* mice (*B*) exposed to O_3_. Abbreviations: av, alveoli; bv, blood vessels; tb, terminal bronchioles. Bars = 100 μm.

**Figure 6:**
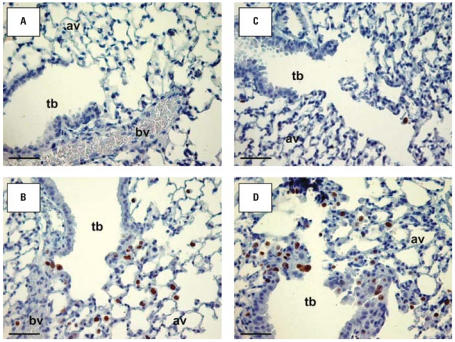
Cell proliferation in lungs of *Mmp9^+/+^* (*A,B*) and *Mmp9*^−/−^ (*C,D*) mice exposed to air (*A,C*) and 48 hr O_3_ (*B,D*). Ki-67–positive cells in G/S phases of the cell cycle were detected immunohistochemically, and representative light photomicrographs display nuclear localization of Ki-67 (brown dots) in cells of severely injured terminal bronchioles. Abbreviations: av, alveoli; bv, blood vessels; tb, terminal bronchioles. Bars = 50 μm.

H&E-stained lung sections from *Mmp9^+/+^* and *Mmp9*^−^*^/^*^−^ mice exposed to air were normal, and no histological differences were found between them (Figure 5A,5C). After 48 hr of O_3_ exposure, significant peribronchiolar inflammation and proliferation as well as epithelial hyperplasia in terminal bronchioles were detected in *Mmp9^+/+^* and *Mmp9*^−/−^ mice (Figure 5B,D). However, O_3_ caused more severe epithelial thickening, inflammation, and alveolar vacuolization in the terminal bronchioles of *Mmp9*^−/−^ compared with *Mmp9^+/+^* mice (Figure 5D). Enhanced airway cellular proliferation was also more severe in the absence of MMP-9 as determined by immunohistochemistry of Ki-67–positive cells in G/S phases of the cell cycle (Figure 6). Nuclear localization of Ki-67 was mainly found in inflammatory cells and in cells of the terminal bronchioles that were injured by O_3_.

### *Effect of* Mmp9 *deficiency on protein levels and mRNA expression of the neutrophil chemokines KC and MIP-2.*

**Figure 7:**
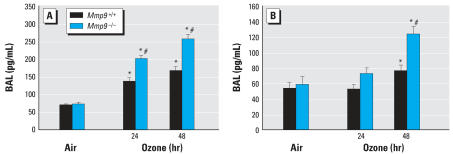
Protein levels of neutrophil chemokines KC (*A*) and MIP-2 (*B*) in BALF from *Mmp9^+/+^* and *Mmp9*^−/−^ mice exposed to air and 0.3 ppm O_3_. Data are presented as means ± SE (*n* = 7–8/group). *Significantly different from genotype-matched air control mice (*p* < 0.05). ^#^Significantly different from exposure-matched *Mmp9^+/+^* mice (*p* < 0.05).

**Figure 8:**
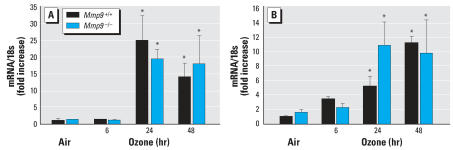
mRNA expression of neutrophilic chemokines KC (*A*) and MIP-2 (*B*) in *Mmp9^+/+^* and *Mmp9*^−/−^ mice exposed to air and 0.3 ppm O_3_. Quantitative real time PCR determined fold increase of message expression normalized to18s rRNA compared with air exposed *Mmp9^+/+^* mice (*n* = 3/group). Data are presented as means ± SE (*n* = 3/group). *Significantly different from genotype-matched air control mice (*p* < 0.05).

To investigate the mechanism for increased neutrophil recruitment to the airway in the absence of MMP-9, we used ELISA to measure BALF levels of chemokines known to be involved in neutrophil chemotaxis (KC and MIP-2). No significant genotype-specific differences in mean concentrations of KC or MIP-2 were found after exposure to air (Figure 7). Mean KC and MIP-2 concentrations were significantly increased in BALF by O_3_ exposure in both genotypes (*p* < 0.05, Figure 7). However, consistent with BALF inflammatory parameters and pathology (Figures 1, 5, and 6), KC and MIP-2 levels were significantly (*p* < 0.05) greater in *Mmp9*^−/−^ mice compared with *Mmp9^+/+^* mice after 24- and 48-hr exposure to O_3_ (Figure 7). KC and MIP-2 mRNA levels were not different between genotypes after exposure to air. After 24- and 48-hr exposure to O_3_, KC and MIP-2 mRNA was increased significantly (*p* < 0.05) over the air controls in both genotypes but, interestingly, no differences were found between genotypes (Figure 8).

## Discussion

O_3_-induced airway inflammation is characterized by early neutrophilic infiltration followed by a mononuclear cell dominated inflammation (Chitano et al. 1995). Molecular changes during the pulmonary pathogenesis induced by O_3_ include increased production of prostaglandins, proinflammatory cytokines, and chemokines such as IL-6, IL-8, granulocyte macrophage colony-stimulating factor, KC, and MIP-2 (Devlin et al. 1991; Johnston et al. 2005a). In humans, neutrophil influx begins in peripheral airways as early as 6 hr after O_3_ exposure and is known to be mediated by neutrophilic chemokines such as IL-8 and GRO-α (Krishna et al. 1998). Functional roles of several inflammatory mediators such as tumor necrosis factor-α (TNF-α) (Cho et al. 2001; Kleeberger et al. 1997), IL-6 (Johnston et al. 2005b), and IL-1β (Arsalane et al. 1995), and the transcription factors nuclear factor kappa B (Cho et al. 2007; Laskin et al. 2002) and activator protein (AP)-1 (Cho et al. 2007) have also been determined in pulmonary inflammation of laboratory rodents exposed to O_3_.

The primary functions of MMPs include degradation and turnover of ECM, tissue repair and remodeling, and leukocyte migration from peripheral circulation to inflammatory sites. Furthermore, recent findings suggested that MMPs can modulate inflammation and innate immunity by affecting the activity of various nonmatrix proteins (Parks et al. 2004). MMP-9 has been thought to be particularly important in the pathogenesis of inflammatory lung diseases, including acute lung injury, asthma, and COPD (Atkinson and Senior 2003). Higher than normal MMP-9 levels in the patients with these disorders may promote destruction of normal tissue architecture and increased migration of inflammatory cells to the disease sites (Lemjabbar et al. 1999a, 1999b).

In the present study, we hypothesized that MMP-9 is essential for O_3_-induced airway inflammation because MMP-9 is one of the most predominant MMPs found in inflammatory airway diseases (Atkinson and Senior 2003). Contrary to predictions, we found that O_3_-induced neutrophilic airway inflammation and injury were markedly increased in *Mmp9*^−/−^ mice compared with *Mmp9^+/+^* mice, indicating a protective role of MMP-9 in O_3_-exposed airways. The results from the current study suggest that MMP-9 may not act primarily on ECM degradation that facilitates leukocyte migration to pulmonary inflammatory sites as indicated by another investigation (Betsuyaku et al. 1999). Heightened neutrophilic inflammation was also associated with enhanced levels of KC and MIP-2, which are important chemokines for neutrophil recruitment to the lung.

MMP-9 has diverse effects on neutrophilic inflammation in experimental animal models. It enhances neutrophil chemotaxis in response to certain chemokines (D’Haese et al. 2000) and increases neutrophil influx in glomerulonephritis (Sternlicht and Werb 2001). Conversely, MMP-9 also has inhibitory effects on BALF neutrophilia (Lanone et al. 2002); increased tissue neutrophil and inflammatory cell infiltration have been shown in *Mmp9*^−/−^mice in response to epithelial injury and chemokine administration (D’Haese et al. 2000; Mohan R et al. 2002). Several lines of molecular evidence have determined that proteolytic function of MMP-9 affects cytokine and chemokine levels as well as their activities. For example, MMP-9 processes and activates pro-IL-1β (Schonbeck et al. 1998), inactive membrane bound forms of TNF-α (Mohan MJ et al. 2002), and transforming growth factor-β (TGF-β) (Yu and Stamenkovic 2000). MMP-9 also processes CXC chemokines, which exert potent chemoattractant activities on leukocytes and alters their specific activities, but not CC chemokines (e.g., RANTES and MCP-2) (Van den Steen et al. 2000). MMP-9 truncates IL-8 (1–77) into IL-8 (7–77), which enhances neutrophil activation more than 10-fold (Van den Steen et al. 2000). In contrast, neutrophilic chemoattractant activity was decreased by MMP-9 degradation of the other CXC chemokines GRO-α and connective tissue–activating peptide (CTAP)-III (Van den Steen et al. 2000). Our current observation that O_3_ induced higher levels of BAL CXC chemokines KC and MIP-2 in *Mmp9*^−/−^ mice than in *Mmp9^+/+^* mice is consistent with, but does not prove, the notion that these chemokines were key effectors of pulmonary MMP-9. Interestingly, we also found no differences in the steady state mRNA levels of the O_3_-increased KC and MIP-2 between *Mmp9^+/+^* and *Mmp9*^−/−^ mice. Together these results suggest that differences in these chemokine levels in *Mmp9*^−/−^ mice compared with those in *Mmp9^+/+^* mice were caused by translational or posttranslational processes, which may include degradation/cleavage by MMP-9.

Significantly greater elevation of MMP-2 level and activity were observed in *Mmp9*^−/−^ mice compared with *Mmp9^+/+^* mice after O_3_ exposure. We cannot rule out the possibility that increased MMP-2 is a compensatory response in *Mmp9*^−/−^ mice and may be involved in increased neutrophilic airway inflammation. However, MMP-9 has been reported to be the dominant airway MMP controlling inflammatory cell egression (Corry et al. 2004), and there is no evidence that MMP-2 has a modulating effect on inflammatory chemokines such as KC and MIP-2 (Parks et al. 2004). In addition, neutrophil concentrations in BALF were not changed in *Mmp2*^−/−^ mice in a model of allergic asthma (Corry et al. 2002). We therefore postulate that MMP-2 does not have a critical role in heightened neutrophilic inflammation in *Mmp9*^−/−^ mice, although it may be involved with other mechanisms of lung injury induced by O_3_ exposure (e.g., lung hyperpermeability).

The role of MMP-7 in acute lung injury has been studied in mouse models of interstitial pulmonary diseases such as fibrosis: *Mmp7*^−/−^ mice had suppressed pulmonary fibrosis caused by bleomycin (Li et al. 2002; Zuo et al. 2002), and it was accompanied by decreased neutrophilic inflammation and chemokines (e.g., KC) in the alveolar fluid (Li et al. 2002). We predicted that O_3_-induced airway inflammation would be attenuated in *Mmp7*^−/−^ mice. However, the current findings suggest that MMP-7 is not significantly associated with O_3_-induced airway inflammation and injury in mice. In addition, KC concentrations in BALF were not significantly different between *Mmp7*^−/−^ and *Mmp7^+/+^* mice in the current model (data not shown). No studies have investigated the role of MMP-7 in oxidative lung injury such as O_3_. Different from MMP-9, matrilysin is produced by alveolar epithelial cells in injured lungs, and it has been thought to contribute to alveolar epithelial injury and re-epithelialization. Inhaled O_3_ mostly affects centriacinar legions of the airway, but not alveoli, which may partially explain why MMP-7 deficiency failed to alter airway inflammation by O_3_.

In summary, our results showed that deficiency in MMP-9 was associated with enhanced airway epithelial injury, neutrophil recruitment, and permeability following O_3_ exposure, but a deficiency in MMP-7 did not significantly affect O_3_-induced airway injury. The aberrant neutrophil recruitment was correlated with increased levels of KC and MIP-2 protein, but not mRNA expression, in the *Mmp9*^−/−^ mice relative to *Mmp9^+/+^* mice. Results are consistent with the hypothesis that enhanced O_3_-induced injury in *Mmp*^−/−^ mice is related to a difference in posttranscriptional processing of these CXC chemokines in the airway. These findings increase our understanding of the pathophysiological process of O_3_-induced lung injury and suggest that MMP-9 produced in the lung in response to oxidative stimuli may have a beneficial function.

## Figures and Tables

**Table 1:** Effect of *Mmp7* deficiency on BALF protein and cell differentials after O_3_ exposure (0.3 ppm for 48 hr).

Genotype	Proteins (μg/mL)	Macrophages (×10^3^/mL)	Neutrophils (×10^3^/mL)	Epithelial cells (×10^3^/mL)
*Mmp7*^+/+^ (Air)	107.0 ± 6.7	21.8 ± 2.8	0.1 ± 0.1	1.7 ± 0.2
*Mmp7*^−/−^ (Air)	140.0 ± 4.7	23.5 ± 1.9	0.02 ± 0.02	1.5 ± 0.3
*Mmp7*^+/+^ (O_3_)	540.8 ± 32.1*	47.6 ± 2.2*	5.9 ± 1.5*	3.0 ± 0.5
*Mmp7*^−/−^ (O_3_)	707.5 ± 73.1*	49.7 ± 4.4*	10.0 ± 3.3*	7.1 ± 1.2*

Results are the means ± SE for 5–8 mice in each group.

* Significantly different from genotype-matched air control mice (*p* < 0.05).
